# Association of Increased Circulating Catecholamine and Glucocorticoid Levels with Risk of Psychological Problems in Oral Neoplasm Patients

**DOI:** 10.1371/journal.pone.0099179

**Published:** 2014-07-21

**Authors:** Huixu Xie, Bo Li, Li Li, Xiao-li Zou, Cai-rong Zhu, Yi Li, Ning Gao, Qianming Chen, Longjiang Li

**Affiliations:** 1 West China School of Stomatology, Sichuan University, Sichuan Chengdu, China; 2 State Key Laboratory of Oral Diseases, West China Hospital of Stomatology, Sichuan University, Chengdu, China; 3 Department of medical record management, West China Second University Hospital, Chengdu, China; 4 West China school of Public health, Sichuan University, Chengdu, China; Weill Cornell Medical College Qatar, Qatar

## Abstract

**Background:**

Noradrenergic pathways and glucocorticoid-mediated signal pathways have been implicated in the growth and progression of oral cancer. Patients with oral neoplasms can have high psychological distress levels, but the effects of stress-related hormones on oral neoplasm growth are unknown.

**Methods:**

We have investigated the relationships between pre-surgical measurements of psychological problems with Symptom Checklist-90-revised Inventory (SCL90-R), tumor histology, circulating blood catecholamine and glucocorticoid levels among 75 oral neoplasm patients, including 40 oral cancer patients and 35 benign oral tumor patients.

**Results:**

The results showed that most dimension scores of SCL90-R did not show a significant difference between the two groups except depression (p = 0.0201) and obsessive-compulsion (p = 0.0093), with the scores for these symptoms being higher among oral cancer group versus the benign oral tumor group. The differences of total score, average score and other monomial factor scores were not statistically significant. The mean concentrations of catecholamine and glucocorticoid in peripheral blood of the oral cancer group were higher than those in benign oral tumor group (p<0.01). We also examined whether associations observed between biobehavioral measures and circulating blood catecholamine and glucocorticoid levels extended to other compartments in the oral cancer group.

**Conclusions:**

These findings suggest that stress hormones may affect oral cancer behavior by influencing the tumor micro-environment though the circulating blood.

## Introduction

A growing number of studies have indicated that the immune alterations resulting from chronic stress and other behavioral conditions may influence cancer development and progression [1]–[3]. Chronic stress is associated with dysregulation of the hypothalamic–pituitary–adrenal (HPA) axis, with consequent increase in the production of the hormone cortisol, and elevated levels of norepinephrine (NE) and epinephrine (E), which are catecholamines released from the adrenal medulla and the neurons of the sympathetic nervous system (SNS) [2], [4]. A key component of the stress response involves activation of the sympathetic nervous system (SNS) and production of mediators which arise both from the SNS and the adrenal medulla [5]. Animal-based research has shown that stress can increase levels of intratumoral NE as well as NE in the ovary and other organs that are typical metastatic sites for ovarian cancer such as spleen and omentum [2], [6], [7].

Patients with oral cancer can have high psychological distress levels, but the effects of stress-related hormones on oral cancer cells and possible mechanisms underlying these relationships are unknown. In one study, the effects of stress-related hormones on interleukin-6 (IL-6) secretion and proliferation of oral squamous cell carcinoma (OSCC) cells was investigated. The effects of NE and cortisol were studied and it was shown that NE and isoproterenol significantly enhanced IL-6 mRNA expression and protein production in SCC9 and SCC25 cells. Physiological stress levels of NE and isoproterenol elicited the most robust IL-6 increase at 1 h. The effects of cortisol varied according to the hormone concentration. These findings suggest that stress hormones can affect oral cancer cell behavior [8].

Noradrenergic pathways have been implicated in the growth and progression of ovarian cancer. Intratumoral NE has been shown to increase with stress in an animal cancer model, but little is known regarding how tumor NE varies with disease stage and with biobehavioral factors in ovarian cancer patients [9]. These results suggest that tumor NE provides distinct information from circulating plasma concentrations. Tumor NE levels were elevated in correlation to tumor grade and stage. Low subjective social support was associated with elevated intratumoral NE. As beta-adrenergic signaling is related to key biological pathways involved in tumor growth, these findings may have implications for patient outcomes in ovarian cancer [9]. In another cancer type, glucocorticoids suppressed androgen-independent prostate cancer growth possibly due to the inhibition of tumor-associated angiogenesis by decreasing VEGF and IL-8 production directly through the glucocorticoid receptor *in vivo* [7].

Gu *et al* [10] used two HPLC-MS-MS (High Performance Liquid Chromatography-tandem Mass spectrometry-Mass spectrometry) methods using the Multiple Reaction Monitoring (MRM) acquisition mode for quantitative analysis of 13 compounds in the catecholamine biosynthetic and metabolic pathways. In contrast to other existing methods, the time consuming sample pretreatment procedure was shortened and even isomeric compounds could be satisfactorily resolved by using the MRM mode. HPLC-MS-MS methods are simple, rapid, reproducible and efficient, and their application might be extended toward other biological samples such as plasma and urine.

In this study, we have evaluated the association between psychological problems and stress-related hormones in primary oral cancer patients. We also examined the relationship between catecholamine levels and tumor stage, which has not been previously characterized. We hypothesized that advanced stage oral cancer would be more associated with higher catecholamine and glucocorticoid levels in circulating blood than worsened psychological problems.

## Methods

### Ethics statement

This study was conducted according to the guidelines laid down in the Declaration of Helsinki and the Ethics Committee of West China Hospital of Stomatology. Sichuan University approved all procedures involving human subjects and animal studies were conducted in compliance with animal care regulations and applicable national laws (research permit: WCHSIRB-D-2013-054). Written informed consent was obtained from all participants in this study.

### Participants

Patients over 18 years of age with a new diagnosis of an oral mass or ulcer suspected to be an oral tumor were recruited. Inclusion was confirmed by histologic diagnosis of primary oral tumor. Patients were excluded for primary cancer of another organ site, oral tumors of systemic disease, use of systemic corticosteroid medication in the previous 6 months, chronic use of beta-blockers, lack of clear histological diagnosis after operation, or comorbidities known to alter the immune response. A total of 75 patients (49 men and 26 women) with oral tumors were included. All tumor samples of included patients were verified by a pathologist. All patients were informed of their diagnosis at the pre-operative conversation. Psychosocial questionnaires were completed during the pre-operative appointment. Blood samples were taken in the pre-surgical waiting area approximately 2 h before surgery, between approximately 9∶00 and 11∶00 AM.

### Biobehavioral, demographic, and medical assessments

Demographic, health behavior, clinical information and histopathologic information was obtained from medical records. Information on demographic characteristics and health behaviors such as hours of sleep, smoking, alcohol and caffeine intake during the 7 days before surgery were provided by patient health reports.

All subjects received the Hopkins Symptom Checklist 90-revised Inventory (SCL90-R), a comprehensive self-assessment survey of 90 questions, which rates a broad range of psychiatric symptoms over the previous 7 days on a 5 point scale, and a demographic questionnaire [11]. In the SCL90-R, subjects responded on a five point scale of distress ranging from “not at all” (0) at one pole to “a little bit” (1), “moderately” (2), “quite a bit” (3) and “extremely” (4) at the other pole [12]. The 90 items of the SCL90-R are grouped along ten symptom dimensions reflecting broad psychological symptom status in a spectrum of individuals. The dimension “psychoticism” (10 items) includes items indicative of a withdrawn, isolated, schizoid lifestyle as well as items representing symptoms of psychosis and schizophrenia such as hallucinations and thought broadcasting. The SCL90-R has shown good internal consistency and test–retest reliability [11]. However, the factor structure of the test has shown inconsistent results. Commonly, less than nine factors are identified, and the psychoticism dimension has been shown to yield the least consistent results [12]. Such inconsistencies have been reported since the very first stages of development of SCL90-R [11]. The SCL90-R inventory measures a broad range of psychological problems and symptoms of psychopathology through three Global Indexes (Global Severity Index, Positive Symptom Distress Index and Positive Symptom Total) and nine primary symptom dimensions comprising a total of 83 items (Somatization, Obsessive-Compulsive, Interpersonal Sensitivity, Depression, Anxiety, Hostility, Phobic Anxiety, Paranoid Ideation and Psychoticism). The Global Severity Index, which is the participant’s mean score (using all 90 items), is a widely used global index of distress.

### Measurement of catecholamines and glucocorticoids

Catecholamine levels were determined by high performance liquid chromatography with Mass spectrometry (HPLC-MS-MS). Plasma samples were collected in chilled EDTA Vacutainer tubes (BD Biosciences, Franklin Lakes, NJ), kept on ice prior to centrifugation, and all samples were stored at −80°C after collection.

Before the sample pretreatment, ascorbic acid was added to the plasma (50 mg/ml) as a stabilizer. Then, 0.1 mL plasma samples were pipetted into a microcentrifuge tube. Acetonitrile (0.1 ml) was added to the sample solution and samples were vortexed for 5 min. After centrifugation at 15000×g for 5 min, the supernatants were introduced into the HPLC-MS-MS system for analysis.

### High performance liquid chromatography (HPLC)

Sample analysis was carried out in an Ultimate3000 HPLC system (Dionex). The separation of analytes was performed on a HaloTM C18 column (2.1×100 mm, 2.7 µm). The mobile phase was a mixture of methanol-0.01% formic acid (v:v = 90∶10). The flow rate was 100 µl/min. The column temperature was set at 25°C.

### Mass spectrometry (MS)

Eluants were introduced into a 3200Q TRAP tandem mass spectrometer (Applied Biosystems) operated in electrospray positive mode (ES+) and multiple reaction monitoring (MRM). The capillary voltage was 5.5 Kv and the source temperature was 550°C. The desolvation gas flow rate was set at 60 L/h. MRM transitions are listed in Table 1.

**Table 1:** MRM transitions of the analytes and their internal standards.

Target compound	Precursor ion (m/z)	Product ion (m/z)
epinephrine	184.1	166.2 135.2 107.1
norepinephrine	170.0	152.2 107.0 135.1
cortisone	361.2	121.3 163.3
cortisol	363.2	121.3 267.3 327.1
D6-epinephrine	190.1	112.2
D6-norepinephrine	176.1	111.2
D4-cortisol	367.2	121.2
D2-cortisone	362.9	165.4

### Statistical analyses

SPSS version 19.0 (SPSS, Inc., Chicago, IL) was used for data analysis. Catecholamine and glucocorticoid concentrations in each compartment were tested for simple association with continuous variables using Pearson correlations and for association with categorical variables using Spearman rank correlations. Following detection of a possible genetic effect on any of the Global Indexes, we compared the mean scores of the nine SCL90-R symptom scales. Chi Squared tests or analyses of variance (ANOVA) for multiple comparisons was performed considering the two different diagnostic subgroups of oral cancer and oral benign tumor on the nine Symptom Subscales. A p value of <0.05 was considered statistically significant.

## Results

### Participant characteristics

Table 2 compares the patient characteristics between the oral benign tumor group and the cancer group. There were no significant associations between age or health behaviors. The comparison between the benign tumor group and the cancer group did not show any differences in age, male proportion, tumor location, income, marital status, educational status, distance to the hospital and time to the hospital. There were no significant associations of relationship status (single vs. divorced/separated/widowed vs. married/living with partner) with catecholamine and glucocorticoid levels in any compartment (p>0.34).

**Table 2:** Patient characteristics.

Characteristics	Benign Group (N = 35)	Cancer Patients Group (N = 40)	t	χ^2^	*P*
Basics					
Age (mean ± Sd)	44.37±14.57	50.7±12.39	2.03	-	0.056
Age (range)	(18, 74)	(27, 78)	-	-	-
Male %	21 (60.0%)	28 (70.0%)	-	0.82	0.364
Income (RMB¥), %					
<1000	7 (20.0%)	8 (20.0%)	-	1.08	0.993
[1000, 2000)	8 (22.9%)	10 (25.0%)			
[2000, 4000)	14 (40.0%)	14 (35.0%)			
[4000, 6000)	6 (17.1%)	7 (17.5%)			
≥6000	0 (0%)	1 (2.5%)			
Marital Status, %					
Single	5 (14.3%)	1 (2.5%)	-	3.84	0.251
Married/living with partner	27 (77.1%)	33 (82.5%)			
Divorced/separated	2 (5.7%)	4 (10.0%)			
Widowed	1 (2.9%)	2 (5.0%)			
Educational Background, %					
Under a high school education	6 (17.1%)	12 (30.0%)	-	4.38	0.345
Senior High School education	9 (25.7%)	9 (22.5%)			
University degree	19 (54.3%)	19 (47.5%)			
Master degree or above	1 (2.9%)	0 (0%)			
Accessibility					
Distance to the hospital (Km)	131.94±156.61 (M = 60.0)	171.35±280.20 (M = 70.0)	−0.18	-	0.861
Time to the hospital (Hr)	3.17±2.68 (M = 2.0)	3.31±3.39 (M = 2.0)	−0.37	-	0.710
Grade					
I	-	9 (22.5%)	-		
II		18 (45.0%)			
III		13 (32.5%)			
Stage					
1	-	4 (10.0%)	-		
2		19 (47.5%)			
3		10 (25.0%)			
4		7 (17.5%)			
Scale Score					
SCL-90(Global Severity Index)	132.46±27.80	143.15±25.12	1.75	−	0.084

### Factor analysis identifying SCL90-R symptom dimensions

Most dimension scores of SCL90-R did not show a significant difference between the benign tumor and cancer patient groups except depression (p = 0.0201) and obsessive-compulsion (p = 0.0093). There were no significant associations of relationship status with somatization (p = 0.1111), interpersonal sensitivity (p = 0.1398), anxiety (p = 0.0962), hostility (p = 0.6099), phobic anxiety (p = 0.5066), and paranoid ideation (p = 0.2734), or psychosis (p = 0.3571) between the two groups (Table 3).

**Table 3:** Comparison of scores of SCL 90 between oral cancer and oral benign tumor groups.

SCL-90	Benign Group (N = 35)	Cancer Patients Group (N = 40)	Z	P
	Mean± Sd	Mean± Sd		
somatization	1.52±0.49	1.60±0.38	−1.59	0.1111
obsessive-compulsion	1.57±0.40	1.82±0.38	−2.60	0.0093
sensitive	1.44±0.39	1.54±0.35	−1.48	0.1398
depression	1.42±0.32	1.57±0.31	−2.32	0.0201
anxiety	1.45±0.30	1.58±0.36	−1.66	0.0962
hostility	1.62±0.60	1.62±0.44	−0.51	0.6099
phobic anxiety	1.43±0.42	1.47±0.37	−0.66	0.5066
paranoid ideation	1.47±0.37	1.54±0.34	−1.10	0.2734
psychosis	1.42±0.34	1.46±0.30	−0.92	0.3571

### Catecholamines, glucocorticoids and tumor histology

**Figure 1:**
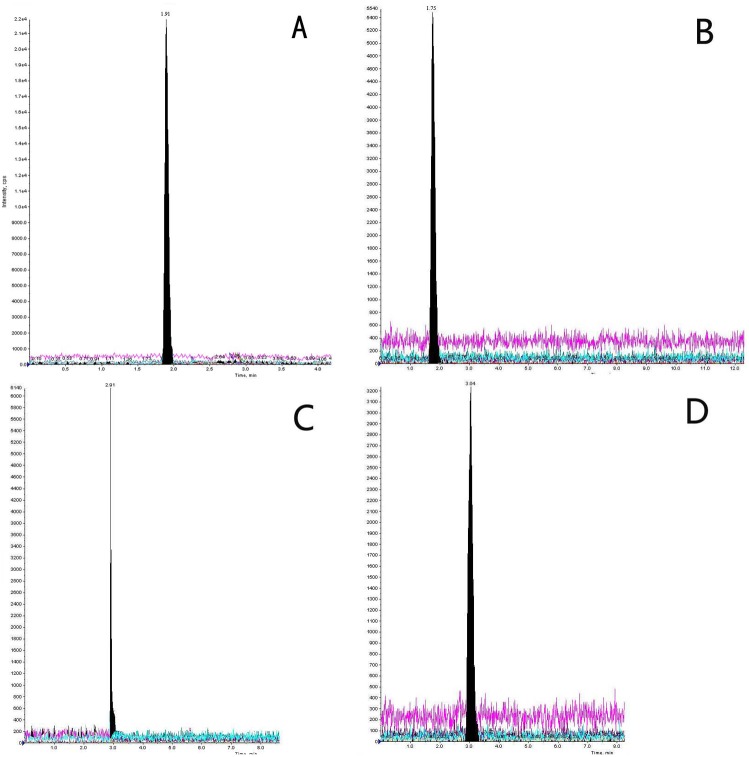
Chromatograms of Adrenaline, Noradrenaline, Cortisone and hydrocortisone.

**Figure 2:**
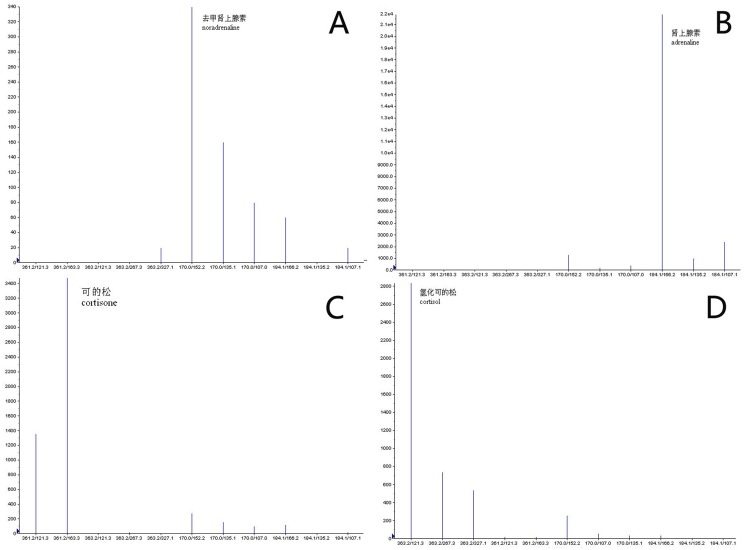
Mass Spectrum of Adrenaline, Noradrenaline, Cortisone and hydrocortisone.

The HPLC-MS-MS method provides better specificity for analysis in comparison to LC-MS, since it monitors ion transitions using MRM. The ions selected for the identification of each analyte were not always the most abundant but were the most specific ones. Representative extracted ion chromatograms and mass spectrum of standard mixture for each compound are depicted in Fig. 1 and 2, respectively.

The mean concentrations of catecholamines in peripheral blood in the oral cancer group were higher than those in oral benign tumor group. The concentrations of catecholamines in peripheral blood were 70.27±34.50 pg/ml (epinephrine) and 316.73±109.22 pg/ml (norepinephrine) in oral cancer group, versus 49.48±31.04 pg/ml (epinephrine) and 252.14±81.80 pg/ml (norepinephrine) in the oral benign tumor group, with the differences between the two groups being statistically significant (all P<0. 01). The mean concentrations of glucocorticoids in peripheral blood in the oral cancer group were also higher than those in the oral benign tumor group. In the oral cancer group, the concentrations of glucocorticoids in peripheral blood were 20.21±7.11 ng/ml (cortisone) and 119.89±44.13 pg/ml (hydrocortisone) in the oral cancer group, versus 8.96±3.09 ng/ml (epinephrine) and 70.44±24.91 ng/ml (hydrocortisone) in oral benign tumor group, with these differences between the two groups also beingstatistically significant (all P<0.001; Table 4).

**Table 4:** Comparison catecholamine and glucocorticoid concentrations between oral cancer and benign oral tumor patients.

Blood index	Benign Group (N = 35)	Cancer Patients Group (N = 40)	*t*	*Z*	*P*
	Mean± Sd	Mean± Sd			
Plasma epinephrine (pg/ml)	49.48±31.04	70.27±34.50	-	−2.97	0.0030
Plasma norepinephrine (pg/ml)	252.14±81.80	316.73±109.22	-	−2.89	0.0039
Plasma cortisone (ng/ml)	8.96±3.09	20.21±7.11	9.07	-	<.0001
Plasma hydrocortisone (ng/ml)	70.44±24.91	119.89±44.13	6.07	-	<.0001

**Figure 3:**
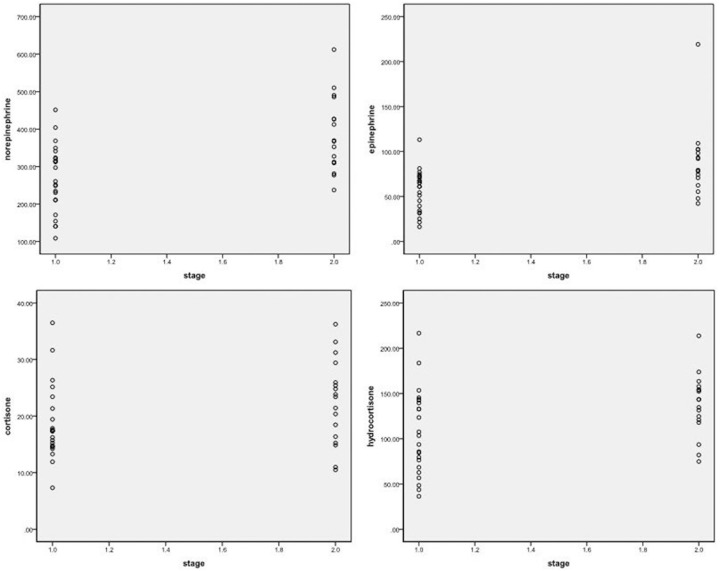
Peripheral blood catecholamines and glucocorticoids levels differed substantially as a function of cancer stage.

Peripheral blood catecholamines and glucocorticoid levels differed substantially as a function of cancer stage, with Stage I and II (early stage) cancer showing comparatively low concentrations (epinephrine 56.61±23.09 pg/ml, norepinephrine 267.81±89.77 pg/ml, cortisone 18.55±6.48 ng/ml, and hydrocortisone 106.95±46.71 ng/ml), and Stage III and IV (advanced stage) cancer showing substantially greater concentrations (epinephrine 88.75±39.20 pg/ml, norepinephrine 382.91±99.50 pg/ml, cortisone 22.44±7.51 ng/ml, hydrocortisone 137.40±34.34 ng/ml) as shown in Figure 3, Table 5. In each case, except for cortisone, the contrast between advanced-stage and early-stage cancer in these measurements was highly significant (Table 5).

**Table 5:** Peripheral blood catecholamine and glucocorticoid levels differed with cancer stage.

Variable	Stage	N	Mean	Std Dev	t	*p*
epinephrine	early	23	56.60	23.09	−3.02	0.006
epinephrine	advanced	17	88.75	39.20		
Norepinephrine	early	23	267.81	89.77	−3.83	0.0005
Norepinephrine	advanced	17	382.91	99.50		
cortisone	early	23	18.55	6.47	−1.76	0.0868
cortisone	advanced	17	22.44	7.51		
hydrocortisone	early	23	106.95	46.71	−2.27	0.029
hydrocortisone	advanced	17	137.40	34.34		

### Biobehavioral factors and catecholamines in three compartments

To better understand the contributions of catecholamines and glucocorticoids in the process of tumorigenesis among patients, we further analyzed possible interactions between these substances and patient mental health characteristics. Patients with depression and/or obsessive-compulsive psychological problems typically had significantly higher catecholamine and glucocorticoid levels in the oral cancer group versus the oral benign group, and levels in the advanced stage cancers were significantly higher than in early-stage cancers.

**Figure 4:**
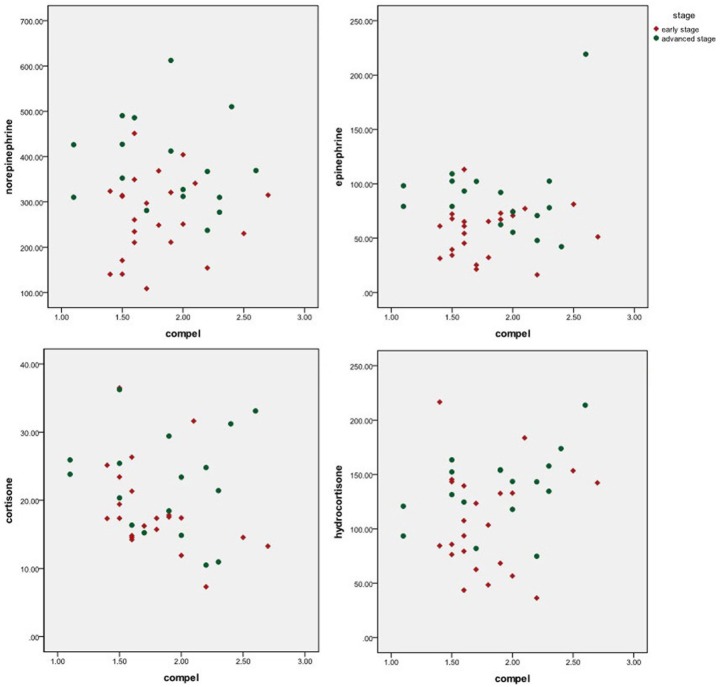
Peripheral blood catecholamines and glucocorticoids as a function of perceived obsessive-compulsive psychological problems for early and advanced stage oral cancer.

As shown in Fig. 4, patients with higher levels of obsessive-compulsive psychological problems had higher peripheral blood catecholamines and glucocorticoids (β^NE^ = 31.364, PNE = 0.039; β^E^ = 115.783, P^E^ = 0.007; βc^ortisone^ = 4.165, P^cortisone^ = 0.071; β^hydrocortisone^ = 28.165, P^hydrocortisone^ = 0.044). We also examined whether the relationships between depression and/or obsessive-compulsive psychological problems and cancer stage would be reflected in the peripheral blood catecholamine and glucocorticoid levels. As with the relation to cancer stage, a similar trend was seen for psychological problems in relation to peripheral blood catecholamines and glucocorticoids.

## Discussion

Clinical and epidemiological studies have demonstrated positive associations between stress and cancer progression. A key component of the stress response involves the locus ceruleus/norepinephrine (LC/NE) sympathetic system and production of mediators such as NE and E, which arise both from the sympathetic system and the adrenal medulla, suggesting that tumor NE provides distinct information from circulating plasma concentrations [7]. It has been proven that tumors in stressed animals show markedly increased vascularization and enhanced expression of VEGF, MMP2 and MMP9[2]. These data identify β-adrenergic activation as a major mechanism by which behavioral stress can enhance tumor angiogenesis *in vivo* and thereby promote malignant cell growth. These data also suggest that blocking ADRB (Adrenergic Receptor β)-mediated angiogenesis could have therapeutic implications for the management of ovarian cancer. It was recently shown that both NE and E levels are elevated in a sustained fashion in ovarian and other peritoneal tissues in preclinical models of chronic stress [2]. These hormonal increases were related to greater tumor burden, which was mediated by increased angiogenesis [2].

The other major neuroendocrine response to stress is via activation of the HPA axis, consisting of consequent release of the neuropeptides corticotrophin releasing hormone and vasopressin, which stimulate pituitary adrenocorticotropic hormone (ACTH) release. This leads to stimulation of glucocorticoid secretion by the adrenal cortex, which is essential for stress adaptation [13], [14]. Mice undergoing stress show rises in endogenous glucocorticoid levels [15], suggesting that glucocorticoids are involved in stress induced thymic-involution [16]. Plasma glucocorticoid levels in tumor-bearing animals were similar to that of normal mice [17].

A growing number of studies have shown that hormonal and immune alterations resulting from chronic stress and other behavioral conditions may influence cancer development and progression [8]. Chronic stress is associated with dysregulation of the HPA axis and the LC/NE sympathetic system, with a consequent increase in the production of the hormone cortisol and elevated levels of NE and E. Catecholamines were released from the adrenal medulla and the neurons of LC/NE sympathetic system to promote tumor growth and angiogenesis [9].

The key findings of this study are that peripheral blood catecholamine and glucocorticoid levels in primary oral cancer patients are linked to both disease severity and patient psychosocial characteristics. Catecholamines and glucocorticoids were elevated in patients with advanced stage disease and higher grade pathology. Symptoms of mental illness were related to plasma catecholamines. Shahzad demonstrated that behavioral stress increases intratumoral NE in an orthotopic mouse model of ovarian cancer [6]. Also, increases in NE in organs that are typical metastatic sites for ovarian cancer have been reported [2]. Sook *et al* found that the intratumoral catecholamine differences observed affect biological pathways involved in tumor progression such as angiogenesis, invasion, anoikis, transcription pathway activation, etc [18]. Others have shown a possible link between psychosocial distress and increased risk of metastasis [2].

In our study, a higher global severity index was associated with increased psychiatric distress between two groups than Chinese norms. However, the difference between the two groups was not significant. Indeed, our data and those of others suggest that patients diagnosed with oral cancer have more emotional distress than patients diagnosed with oral benign tumors. We observed similar global distress severity among the groups of patients. In the whole study, patients with lower scores on the SCL90R scale were at lower stage than those with higher scores. These findings provide support for linking depression and obsessive-compulsion with oral cancer. However, our data partially support that the circulating levels of catecholamine may be related to cancer stage. Greater psychological problems were not associated with higher catecholamine and glucocorticoid levels in circulating blood versus tumor tissue.

In comparison to other studies, we found high rates of psychotic symptoms in the two groups. Lutgendorf reported elevated tumor catecholamines accompanying biobehavioral risk factors in a small sample of human ovarian cancer patients [19]. It was also reported that tumor NE provides distinct information from circulating plasma concentrations. Tumor NE levels were elevated in accordance with tumor grade and stage. Low subjective social support was associated with elevated intratumoral NE [9]. We found advanced stage oral cancer to be associated with higher catecholamine and glucocorticoid levels in peripheral blood, and our data suggest that those substances affect the tumor micro-environment though the circulating blood rather than the tumor matrix. These findings supported our hypothesis that oral cancer patients would have high levels of peripheral blood catecholamines, whereas oral benign tumor patients would have lower levels. Contrary to our hypothesis, both groups of patients were associated with high levels of psychological problems such as obsessive-compulsion and depression.

The present study has several limitations. First, we did not control for the fact that different surgeries might have affected the patient’s cognitive ability. Further studies including the assessment of objective cognitive impairment will be needed to determine the effect of differences in surgical procedures. Also, this study did not provide practical implications of subjective symptoms. Thus, additional research is necessary to confirm the relationship between subjective symptoms and difficulties in daily lives of individuals with oral cancer.
